# New Mcconnellite Ceramic Pigment as a Selective Solar Absorber: Effects of Microwave Firing and Rare Earth Doping

**DOI:** 10.3390/ma18071520

**Published:** 2025-03-28

**Authors:** Guillermo Monrós, José Antonio Badenes, Carolina Delgado, Guillem Monrós-Andreu, Mario Llusar

**Affiliations:** 1 Department of Inorganic and Organic Chemistry, University Jaume I, 12006 Castelló de la Plana, Castelló, Spain; jbadenes@uji.es (J.A.B.); caroladelg@gmail.com (C.D.); mllusar@uji.es (M.L.); 2 Department of Mechanic Engineering and Construction, University Jaume I, 12006 Castelló de la Plana, Castelló, Spain; gmonros@uji.es

**Keywords:** ceramic pigment, mcconnellite, microwaves, selective solar absorber, ceramic glaze

## Abstract

CuCrO_2_ (mcconnellite) was synthesized using both the solid-state method and microwave dielectric firing. It was characterized as a novel black ceramic pigment for use in various industrial glazes. For the first time, the application of mcconnellite (CuCrO_2_) and its coloured glazes as selective solar absorbers (SSA) for integral ceramic solar collectors has been reported. The addition of quartz or anatase as colour modifiers was investigated to prevent the bluing of the pigment in Zn-containing glazes, a phenomenon associated with the exsolution of copper. Furthermore, doping with lanthanide oxides was explored to address two key challenges: controlling the formation of pinhole defects in porcelain glazes, which are linked to the destabilization of Cu^+^, and adjusting the IR cut-off wavelength to improve its performance as SSA.

## 1. Introduction

Mcconnellite CuCrO_2_ belongs to the ternary oxides ABO_2_ (A^+^ of Cu, Pt, Ag, and B^+3^ of Fe, Cr, Al, Ga) of delafossite class of minerals which show a laminar structure formed by sheets of BO_6_^−9^ octahedrons linked by O-A^+^-O dumbbells. Two polymorphs of delafossite are described in the literature: the hexagonal delafossite (*S.G. P6_3_/mmc*) first reported by Kohler and Jansen in 1983, and the trigonal delafossite (*S.G. R-3m*) described by Pabst in 1939 [1,2]. Cu delafossite families, CuM^IIIA^O_2_ (MIIIA = Al, Ga, In) and Cu^MIIIB^O_2_ (MIIIB = Sc, Y, La) stabilize in rhombohedral and hexagonal l structures, respectively [2,3,4].

The efficient use of thermal energy derived from solar radiation using FPSC (flat-plate solar collector) requires an efficient and low-cost selective solar absorber (SSA) [5]. Based on different dielectric materials, coatings can mainly be categorized into double-cermet solar selective coatings, transition metal nitride multilayer coatings, and transition metal oxide multilayer coatings [6]. Selective absorbers are optimized for either concentrated collectors or flat-plate collectors, where under these conditions, *α* (solar absorptance) has a greater impact than ε(T) (thermal emittance). For high-temperature applications, vacuum insulation in a solar thermal panel minimizes internal gas convective and conductive losses to a negligible level, maintaining high conversion efficiency at elevated working temperatures [7]. Recently, for spectrally selective absorbers (which should efficiently absorb light in the solar spectrum (α = 1) while minimizing thermal radiation emission (ε(T) = 0)), the optimal cut-off wavelength, transitioning from high absorbance to high reflectance (low emittance), is determined by the operating temperature. An increase in operating temperature requires a decrease in the cut-off wavelength [8,9].

Yang et al. [10] reported an integral ceramic solar collector made entirely of ceramics based on a low-porosity ceramics body, through which the heat transfer liquid circulates, and coated with a slurry of a black ceramic pigment as selective solar absorber surface; the ceramic assembly was fired at 1210 °C. The selective solar absorber is a critical component of solar collector, designed to maximize solar absorbance within the solar spectrum range (300–2500 nm) while allowing thermal emission in the infrared range, beyond the solar spectrum limit of approximately 2000 nm [10]: an ideal SSA shows a very sharp spectral transition between the region of high absorbance and low emittance (cut-off wavelength) to the region of low absorbance and high emittance. For energy applications, the cut-off wavelength should be around 2000 nm, which is the limit of the solar spectrum [11]. In a previous paper, Monrós et al. [12] reported the synthesis and characterization of coloured glazes with tetragonal CuCr_2_O_4_ as selective solar absorbers for Integral ceramic solar collectors. The prepared tetragonal CuCr_2_O_4_ shows the presence of mcconnellite CuCrO_2_ as residual phase in all cases showing its high stability [12].

Passive thermal management includes both passive heat dissipation and passive heat recovery from the sun. Passive heat dissipation refers to the natural release of heat generated by a heat source, such as a CPU, into the surrounding air. This process includes natural convection cooling, heat pipe cooling, and phase change heat storage dissipation.

On the other hand, passive solar heat recovery involves the absorption of solar energy using, for example, a selective solar absorber (SSA). In both cases, a liquid cooling system takes advantage of the high heat transfer coefficient of liquid flow to transfer excess heat, which is ultimately carried away by the coolant as it moves through the device’s internal flow channels [13].

Ceramics are among the most affordable engineering materials and are widely used in technical applications. They are particularly suitable for the construction of solar collectors due to their high thermomechanical stability, excellent workability, resistance to thermal stress, low cost, simple and well-known manufacturing, in addition to their long service life while maintaining their absorption capacity. Additionally, ceramics integrate seamlessly into buildings.

The manufacturing process of a solar collector made entirely of glazed ceramic involves preparing the ceramic body with grooves for the passage of the collector fluid. This process requires the formulation of a ceramic paste through grinding in an aqueous medium, followed by shaping and sintering. A coloured glaze containing a black pigment is then applied to the surface, which, after firing at the appropriate glaze temperature, imparts the characteristics of an SSA.

In this communication, mcconnellite CuCrO_2_ is synthetized using both solid-state method and by dielectric calcination using microwaves. It is characterized as a new black ceramic pigment in different industrial glazes. The pigment and its coloured glazes are evaluated as selective solar absorber SSA. To address specific challenges, the addition of colour modifiers, such as quartz or anatase, is explored to mitigate the bluing effect of the pigment in zinc-containing glazes [12], which is associated with copper exsolution. Furthermore, doping with lanthanide oxides is investigated to control pinhole defects in porcelain glazes caused by the destabilization of Cu^+^ ions [14,15]. Studies also examine the cut-off wavelength to enhance the infrared (IR) emittance of the SSA. To the best of our knowledge, the application of mcconnellite (CuCrO_2_) as a ceramic pigment and its use in coloured glazes for selective solar absorption in integral ceramic solar collectors has not been previously documented.

## 2. Materials and Methods

Solid-state or ceramic method of obtaining mcconnellite CuCrO_2_ samples were carried out from tenorite CuO and eskolaite Cr_2_O_3_ oxides as precursors, with a particle size ranging from 0.3 to 5 µm. Likewise, for the mcconnellite-modified samples, additional precursors were used, including quartz (supplied by QUIMIALMEL SA, Castelló de la Plana, Spain, 99.5 wt.% purity) and lanthanide oxides (La_2_O_3_, CeO_2_ and Pr_6_O_11_, 99.9% purity supplied by ALDRCH SA (Darmstad, Hesse, Germany). The precursors were mechanically homogenized in an electric grinder (20,000 rpm) for 5 min and then fired at 1000–1100 °C for 3 h in electric kiln.

Likewise, the mixture underwent dielectric firing using microwave-assisted heat treatment. Microwave-assisted dielectric firing is a considered “fast firing” for the synthesis of solids [16,17]. To significantly enhance the diffusion rate of ceramic precursors (shortening reaction time and potentially lowering reaction temperature) there has been growing interest in microwave-assisted methods. Additionally, microwave-assisted techniques are considered environmentally friendly, as they require less energy than conventional material processing methods. This makes microwave synthesis an example of “green chemistry” or “sustainable chemistry” [18].

The response of materials to microwaves primarily depends on their complex permittivity ε, which describes the combined effects of permittivity and conductivity. Conductivity is represented as the imaginary component of the permittivity. Therefore, the complex dielectric constant ε has a real component (ε′) and an imaginary component (ε″), also known as the dielectric loss factor (ε = ε′ + i ε″). The heat generated in the material–microwaves interaction is directly related to the “loss tangent” (tanδ = tan(ε″/ε′)).

Transparent or microwaves-inactive materials, such as ceramics, have a low loss tangent (tanδ < 0.01) and penetration depth in the order of metres). On the other hand, susceptors, absorbents, or active materials, such as SiC, graphite or water, exhibit a medium loss tangent (tanδ > 0.1) and penetration depth in the order of centimetres. Finally, microwaves-reflective materials, such as metals and conductive materials, exhibit a high loss tangent (tanδ > 100) and penetration depth in the order of micrometres.

However, the dielectric loss of transparent materials increases gradually with temperature in some cases, such as silica or alumina. In contrast, in other materials like titania or zirconia, the tanδ increases significantly at a critical temperature (around 600 °C for titania and 750 for alumina). Therefore, a hybrid microwave-assisted heat treatment of the mixture of solid reactants is possible. This can be achieved by pre-heating in an electric kiln (Blossi et al. [17]), adding a susceptor (SiC, C, Si_3_N_4_) to the mixture to act as an internal auxiliary heater during initial microwave irradiation, or using a closed kiln with its inner surface coated with a susceptor that acts as an external heater during initial irradiation [17]: the susceptor increases in temperature under microwave irradiation and heats the reactant mixture via radiation and/or conduction. Once the mixture reaches the critical temperature, it continues to heat autonomously. 

**Figure 1:**
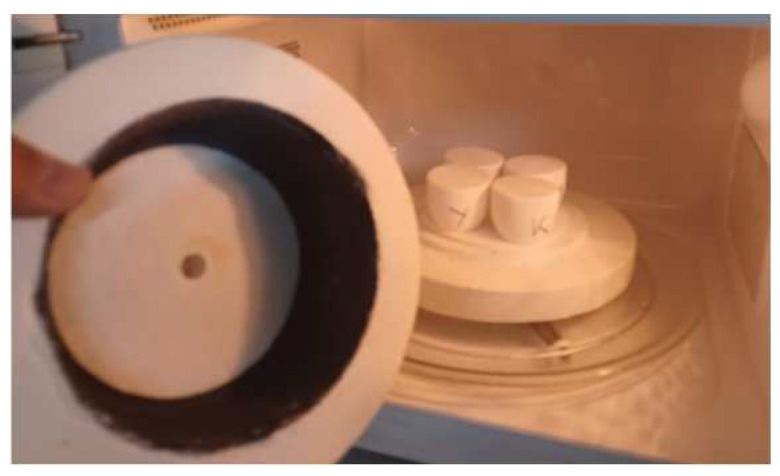
Microwaves pre-heating kiln with the coating of the susceptor.

In this study, a hybrid dielectric firing was carried out using microwave-assisted heat treatment in a conventional microwave equipment operating at 800 W and 2.45 GHz. The equipment included a cylindrical kiln for the pre-heating of the mixture (inner dimensions: height, 4.5 cm; diameter, 13.5 cm) made of aluminosilicate fibreboard (0.3 g/cm^3^, transparent to microwaves; penetration depth of 0.007 m at room temperature and 0.0025 m at 1300 °C). The kiln’s interior was coated with SiC as susceptor (α-SiC hexagonal, density: 3.2 g/cm^3^, penetration depth of 12 m at room temperature and 1.2 m at 1300 °C) (Glass Fusing & Cutting Tools, Tianjin, China, 1282151) (Figure 1).

The temperature was estimated using process temperature control rings (PTCR). Based on PTCR shrinkage measurements after firing (ETH type, 850–1100 °C) with a micrometre, the temperature reached approximately 850 °C at 20 min, 950 °C at 30 min, and 1050 °C at 60 min.

The samples were characterized by the following techniques:

X-ray diffraction (XRD) was performed on a Siemens D5000 diffractometer (Munich, Germany) using Cu K_α_ radiation (10–70°2θ range, scan rate 0.03°2θ, 5 s per step and 40 kV and 20 mA conditions).

Optical microscopy using an Olympus IXplore IX85 microscope (Tokyo, Japan) and scanning electron microscopy with energy-dispersive spectroscopy (SEM–EDS) analysis of X-ray studies using a JEOL 7001 instrument (Tokyo, Japan) were used to analyze the microstructure of the powders.

The L*a*b* and C*h* colour parameters of the glazed samples were measured according to the CIE-L*a*b* (Commission Internationale de l’Éclairage) [19], using an X-Rite SP60 spectrometer (Grand Rapids, MI, USA) with standard lighting D65 and a 10° observer. In this method, L* measures the lightness (100 = white, 0 = black) and a* and b* chromatic components (−a* = green, +a* = red, −b* = blue, +b* = yellow). The CIEL*C*h colour space is highly correlated with colour perception by the human eye; C* represents chroma [19], and h* is the hue angle, which can be estimated from parameters a* and b* using Equations (1) and (2), respectively.

C* = (a^2^ + b^2^)^1/2^ (1)

h* = arctan (b*/a*)(2)

The tolerance ΔE* (based on the L*a*b* parameters), is evaluated using Equation (3):

(3) $${\Delta E}^{*}=\sqrt{{\Delta L*}^{2}+{\Delta a*}^{2}+{\Delta b*}^{2}}$$

UV-Vis-NIR spectra of both the fired powder and glazed samples were collected using a Jasco V670 spectrometer with the diffuse reflectance technique, which provides data in absorbance or reflectance units (R (%)). The spectra are normalized with a blank of the same coating of the integrating sphere (BaSO_4_) for taking into account the lamp response. The band gaps of the samples were estimated using the Tauc method [20]. The total solar reflectance was evaluated from the UV-Vis-NIR spectra using the diffuse reflectance technique, while both absorbance and emittance are estimated using Equations (4) and (5):

(4) $$\alpha =\frac{\int_{400}^{2500}(1-r\left(\lambda \right)i\left(\lambda \right)d\lambda }{\int_{350}^{2500}i\left(\lambda \right)d\lambda }$$

where r(λ) is the spectral reflectance (Wm^−2^) measured by UV-Vis-NIR spectroscopy and i(λ) is the standard solar irradiation (Wm^−2^ nm^−1^) according to the [21]

(5) $$\varepsilon =\frac{\int_{2500}^{20000}(1-r\left(\lambda \right)E\left(\lambda ,T\right)d\lambda }{\int_{2500}^{20000}E\left(\lambda ,T\right)d\lambda }$$

where ε is the emittance that measures the ability to release heat that it has absorbed for a material, and therefore, its temperature is moderated [22], r(λ) is the spectral reflectance (Wm^−2^) measured by UV-Vis-NIR spectroscopy and E(λ,T) is the blackbody radiation spectrum at the considered temperature (Wm^−2^ nm^−1^) [23]. The emittance index of solids is high (e.g., ceramics 0.9, asphalt 0.88) except in the case of conductive metals (e.g., Ag 0.02, Al 0.03, Cu 0.04) [12].

The pigmenting capacity of the corresponding pigment was studied by glazing the pigment in three types of glazes (see composition in Table 1): (a) a double-firing frit with maturation point at 1000 °C, (b) a double-firing frit at 1050 °C, and (c) a porcelain single-firing frit at 1190 °C. The glazed samples were prepared using white stoneware tile substrates coated with glaze. The glazed sample was prepared by manual mixing of frit, pigment, and water in a weight ratio of 97:5:40 in an agate mortar and applying it to a thickness of approximately 1500 μm using the doctor blade technique.

Molten glazes attack pigment particles by dissolving or reacting with them, leading to potential degradation or alteration of colour. The aggressiveness of the glaze increases with the maturation temperature and chemical composition, following the sequence a–c. An efficient ceramic pigment should retain its pigmentation capacity, even in aggressive glazes.

The pigmenting capacity of a ceramic pigment in glazes is associated not only with its intense colour and its thermal and chemical resistance to molten glaze attack, but also with its high refractive index, which efficiently scatters light. The refractive index of mcconnellite is reported to range between 1.5 and 2.5 in the visible spectrum, which surpasses recognized opacifiers such zircon or rutile [23]. The ceramic pigment particles should be stably immersed in the glass matrix and the incidence light scattered, showing a particle size in the order of the wavelength and a high refraction index (n). Assuming this, due to the surface irregularity of the powder submitted to the integrating sphere for diffuse reflectance measurements, the specular radiation energy (which would be observed if the surface were perfectly polished and obeyed Snell’s Law and the Fresnel equations) is instead distributed across the entire material surface. This occurs because the incident light is reflected in all directions and the planes perpendicular to the surface.

Since an integrating sphere allows for the measurement of the total energy spread over the entire surface, the total diffuse radiation energy can be assumed to closely approximate the energy that would be reflected specularly if the material were polished. This is because the refractive index is an intrinsic property of the material itself, rather than its surface characteristics. Therefore, the refractive index (n) can be estimated using the equations derived from Fresnel’s and Snell’s laws for the air–material interface:

(6) $$R={\left(\frac{1-n}{1+n}\right)}^{2}$$

where α is the absorbance measured from the diffuse reflectance spectra, and λ is the wavelength of the incident radiation [24,25].

## 3. Results and Discussion

Table 1 shows the estimated compositions by EDS of employed frits above described. All frits were milled to a particle size between 1 and 15 µm. The alumina consistently increases with the maturation temperature of the glaze. On the other hand, the glaze at 1000 °C is ZnO-free, and the porcelain single-firing frit (1190 °C) shows a low amount of ZnO (6 wt.%) compared with the double-firing glaze at 1050 °C (9 wt.%).

### 3.1. Characterization of Powders: Effect of Microwaves Firing

**Figure 2:**
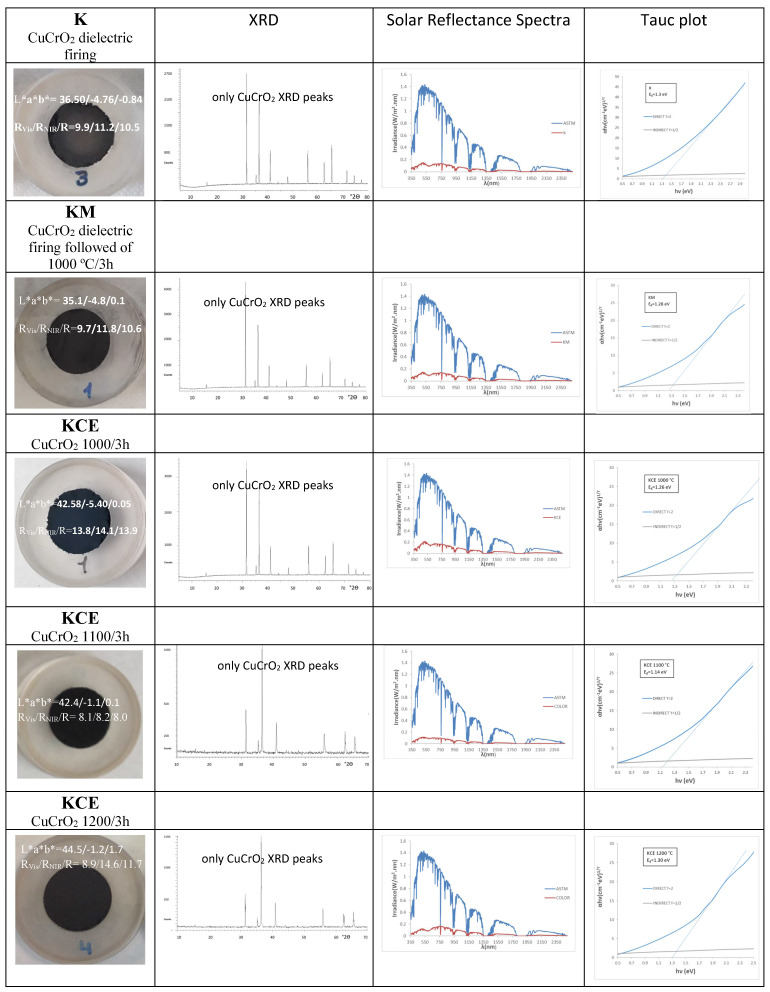
Characteristics of CuCrO_2_ powders: reference commercial carbon black (L*a*b* = 20.2/0.1/0.1, R_Vis_/R_NIR_/R = 3/3/3, C* = 0.14, h = 45).

Figure 2 and Table 2 show the characteristics of black powders K (fired with microwaves for 60 min at 800 W), KM (fired with microwaves followed by firing at 1000 °C for 3 h in an electric kiln), and KCE (fired in electric kiln at successively 1000, 1100, and 1200 °C for 3 h).

**Figure 3:**
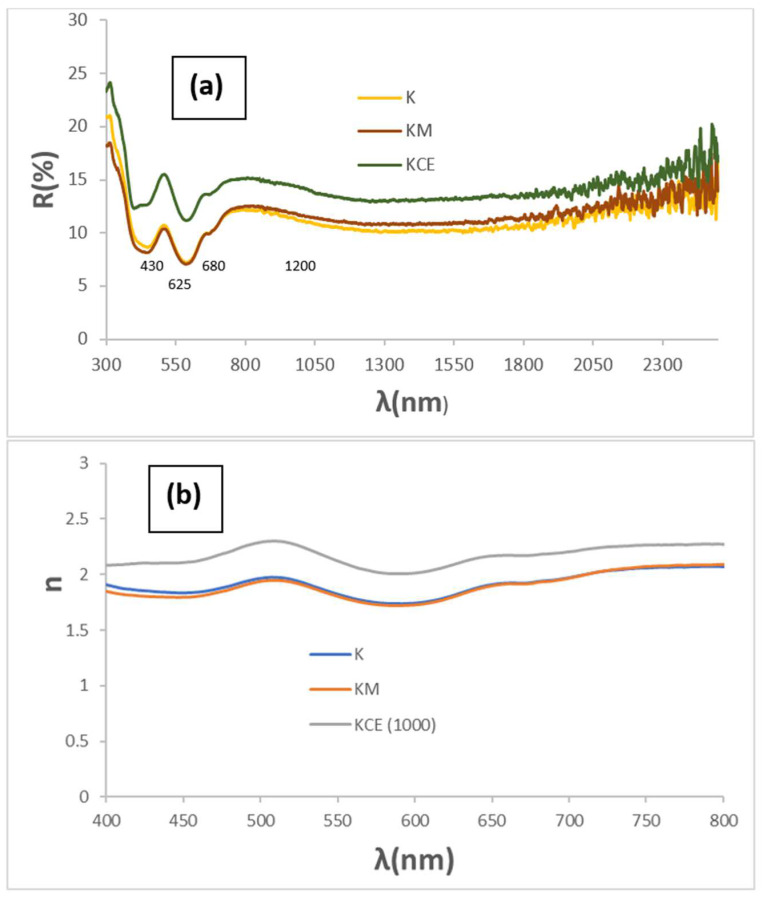

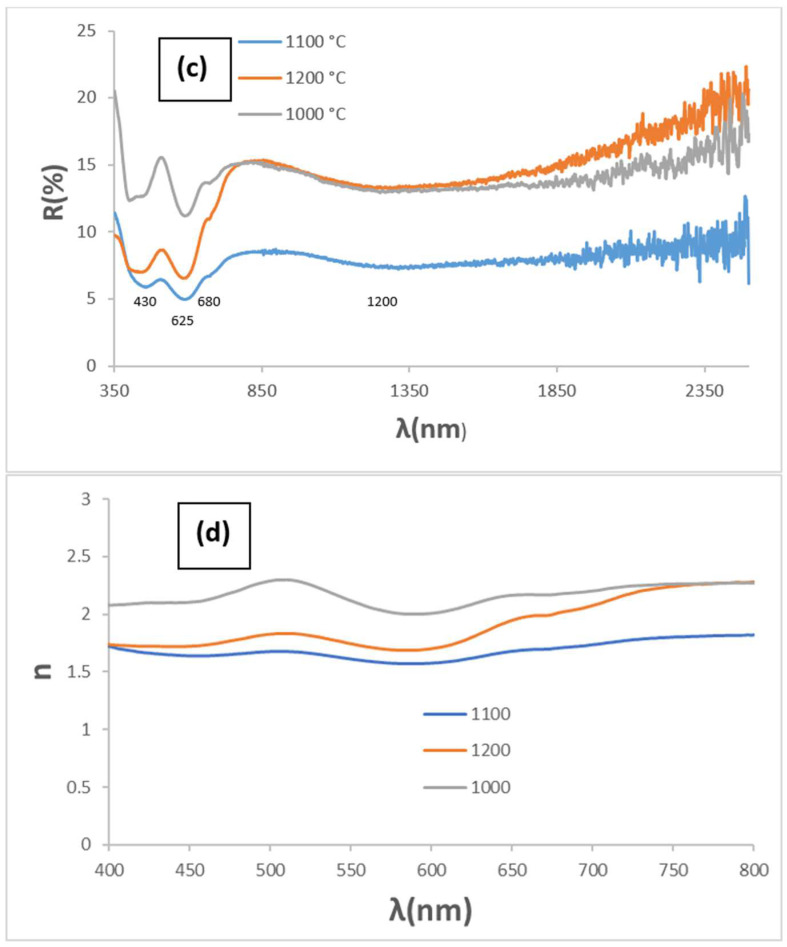
(**a**) UV-Vis-NIR diffuse reflectance spectra of powders K, KM, and KCE (at 1000 °C/3 h), (**b**) refractive index (n) of samples a. (**c**) UV-Vis-NIR diffuse reflectance spectra of powders KCE fired at indicated temperature, (**d**) refractive index (n) of samples c.

The microwaves-assisted sample (K) fired for 60 min at 800 W (~1050 °C), the corresponding KCE (fired at 1000 °C for 3 h), and KM powders exhibit an intense black colour. However, both KM and K show a lower deviation ΔE* from the carbon black used as reference (ΔE* = 15.7 and 17.0 and R_Vis_ = 9.7% and 9.9%, respectively), as shown in Table 1, compared to those fired with an electric kiln (ΔE* = 23.1 and R_Vis_ = 13.8%). Additionally, the extra electric firing of the KM sample does not significantly increase the black shade of powder (R_Vis_ decreases only 0.2%). The band gap of samples measured by the Tauc procedure (see Figure 3 and Table 2) slightly decreases from K (1.30 eV) to KM (1.28) and KCE (1.26 eV), in agreement with the decrease in the black shade (increase in R_Vis_). Figure 2 shows the XRD diffractograms of the powders: in all samples, mcconnellite CuCrO_2_ (JCPDS 039-0247) is the only crystalline phase detected.

Figure 3a shows the corresponding UV-Vis-NIR reflectance spectra of powders: absorbance bands (minima in the figure) at 430, 620, 680, and 1200 nm can be observed as associated with Cr^3+^ (d^3^) absorptions in octahedral environments: (^4^A_2g_(4F) → ^4^T_1g_(4F)) at 430 nm, ^4^A_2g_(4F) → ^4^T_2g_(4F)) detected at 680 nm, and (^4^A_2g_(4F) → ^2^E_g_(2G)) at 1000–1300 nm. The low coordinated Cu^+^ in the O-Cu^+^-O dumbbells of mcconnellite can produce the absorption at 625 nm [19,20]. However, the intensity of these d–d transitions does not explain the anomalously high optical density detected. Therefore, as in the case of the Mn-Melilite and Egyptian Blue pigments, spin–exchange interactions of Cr^3+^ with adjacent Cu^2+^ by a paired spin exchange transition (PET) should be considered [23,25,26]. Figure 3b shows the refractive index of the K, KM, and KCE powders. Both microwave-treated samples exhibit a lower refractive index (n) than KCE. Consequently, KCE scatters light more efficiently, leading to a decrease in absorbance. As a result, R_Vis_ is higher (see Table 2), indicating a slightly lighter black colour.

**Figure 4:**
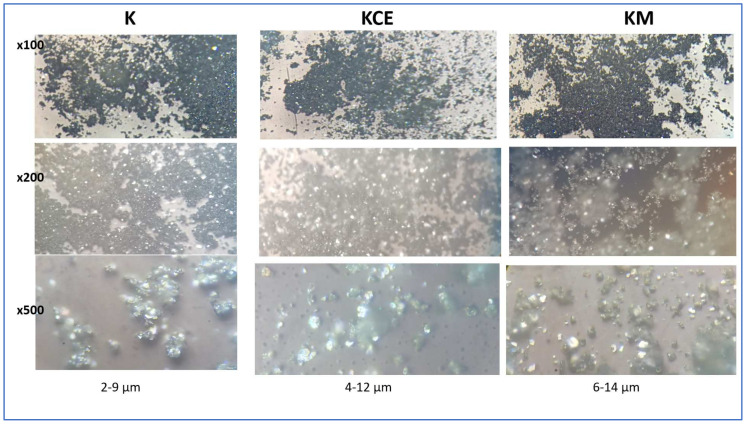
Optical images of CuCrO_2_ samples indicating the estimated particle size at a magnification ×500.

**Figure 5:**
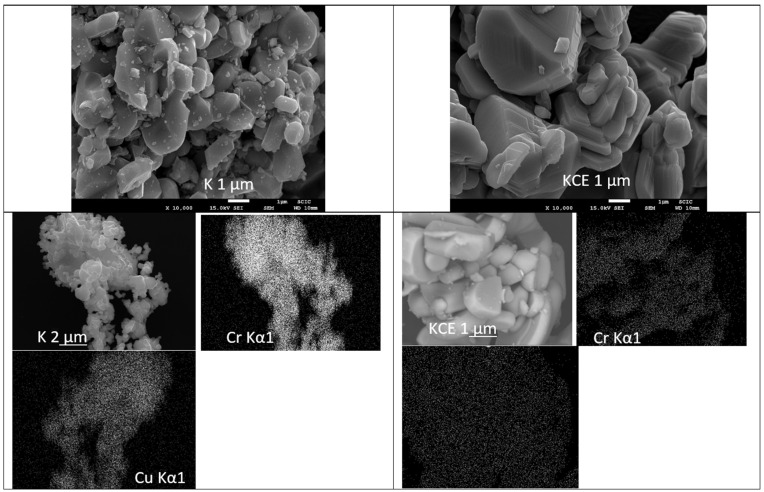
SEM and mapping images of CuCrO_2_ fired by microwaves (K) and by electric kiln 1000 °C for 3 h (KCE).

Figure 4 shows the optical images of these CuCrO_2_ samples; a low particle size is observed for microwave-fired powder (sample K). The SEM images (Figure 5) of KCE 1000 °C/3 h show platelet-shaped particles forming aggregates, with intercalation between them in the range of 5–15 µm. The microwave-fired sample (K) exhibits similar characteristics but with more rounded and finer particles. The respective EDS mapping of K and KCE powders (Figure 5) indicates a homogeneous distribution of Cu and Cr components in both samples. In agreement with the XRD diffractograms, only mcconnellite is observed. In contrast, this phase remains stable in the previously studied CuCr_2_O_4_ formulations and is consistently detected in the synthesis [12].

The small particle size and microstructure of the microwave-fired powders can be associated with its highest black intensity of colour (lowest ΔE* and R_Vis_). Likewise, as discussed above, KCE has a higher refractive index than the microwave-treated powders, allowing it to scatter light more efficiently. However, it was recently found that the use of microwaves improves the reactivity of Cu-involved systems, for example, by improving the degree of Cu doping in the fluorite (Ce-La)O_2_ lattice, which induces a higher catalytic activity in CO oxidation. This increase is associated with the presence of oxygen vacancy sites, together with the well dispersed Cu-O species (which act as a reservoir of Cu active sites for CO adsorption). In fact, the enhanced black colour of the microwave-assisted sample could be associated also with some increase of oxygen vacancy sites or with the better dispersion of Cu ions in the lattice. In any case, this “microwave effect”, widely recognized in organic chemistry [27] and observed in the samples, can be further studied in the future [28].

The increase in firing temperature for KCE powder does not significantly affect the black shade: although both R_Vis_ and chroma C decrease, the lightness (L) of the powders at 1100 and 1200 °C remains above 42, and ΔE remains greater than 22 (Table 2). The UV-Vis-NIR diffuse reflectance spectra of KCE at different firing temperatures, along with the corresponding XRD results, are very similar, and mcconnellite is the only phase detected (Figure 2 and Figure 3). The sample fired at 1100 °C exhibits a slightly better black response compared to those fired at 1000 or 1200 °C. In fact, the estimated refractive index for this sample (Figure 3d) is lower, indicating a slightly reduced opacification power in glazes.

The NIR reflectance is low for all powders, ranging between 8.2 and 14.6% for KCE fired at 1100 °C and 1200 °C, respectively. Similarly, the absorbance and emittance of all these samples are high (Table 2). The cut-off wavelength between the regions of high absorbance and high reflectance is unsharpened, with a soft bandgap ranging from 1.26 eV for KCE fired at 1000 °C and 1.30 eV for the dielectric-fired K sample, which is a limitation for its application as an SSA.

### 3.2. Characterization of Glazed Samples in Double-Firing Glaze at 1000 °C: Effect of Microwave Glazing

**Figure 6:**
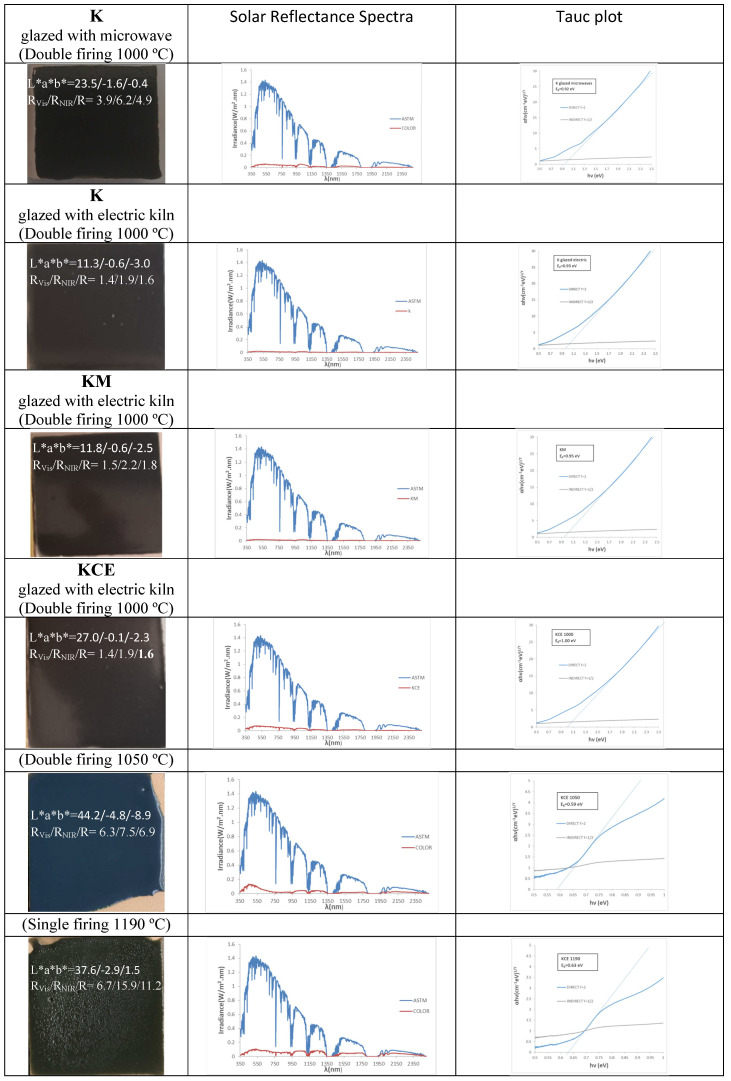
Glazed samples.

Figure 6 and Table 2 show the characteristics of 5 wt.% glazed powders of K (microwave firing), KM (microwave firing followed by electric firing at 1000 °C for 3 h) and KCE (electric firing at 1000 °C for 3 h) in the double-firing glaze at 1000 °C. Likewise, K powder was glazed in the same glaze, but using microwave firing (20 min, 800 W, around 850 °C). Microwave glazing produces a black shade closer to carbon black than conventional glazing with an electric kiln (ΔE* = 3.7 versus 9.5). However, conventional glazing with an electric kiln shows both lower lightness than carbon black (L* = 11.3 versus 20.2 for carbon powder) and visible reflectance (R_Vis_ = 1.4 versus 3.0 for carbon black). Therefore, glazing with a conventional electric kiln, the sample calcined with microwaves produces better black colour than the observed for the carbon black powder used as colour reference, absorbing a 98.6% of visible light. The glazed sample of the KM powder (subjected to 60 min of dielectric firing and then fired in the electric kiln) shows a black shade behaviour very similar to that of the KCE sample (R_Vis_ = 1.4 and ΔE* = 8.8). Therefore, the electric firing following microwave treatment does not significantly improve the final result.

**Figure 7:**
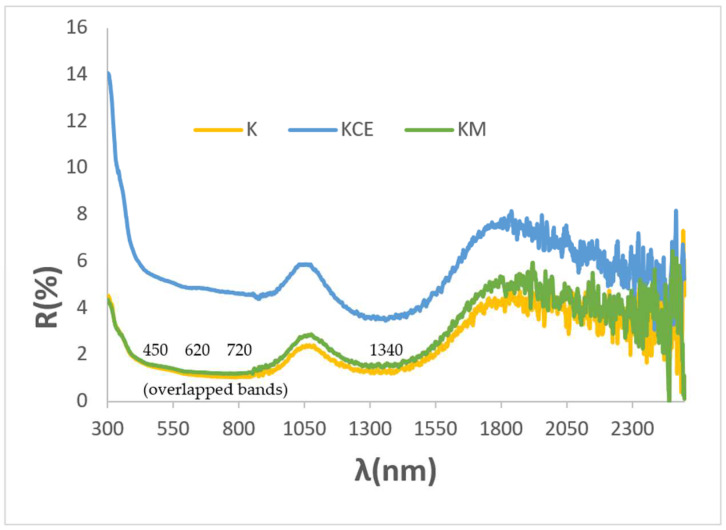
UV-Vis-NIR diffuse reflectance spectra of glazed samples in double-firing frit 1000 °C.

Figure 7 shows the UV-Vis-NIR diffuse reflectance spectra of the glazed samples in double firing frit 1000 °C. All samples exhibit similar spectra, with bands located at similar positions as those in the powder samples: bands at 450, 720 and 1340 nm are detected, associated with Cr^3+^ (d^3^) absorptions in octahedral environments as previously described, although they shift to higher wavelength. The low-coordinated Cu^+^ in O-Cu^+^-O dumbbells of mcconnellite can produce an absorption at 620 nm which overlaps with the bands of Cr^3+^ (d^3^) at 450 and 720 nm. Consistent with their similar optical spectra, the bandgap measured from the Tauc plots shown in Figure 6 are very similar: 0.92, 0.93, 0,95, and 1.00 eV for K (microwave firing and glazing), K (microwaves glazed with electric kiln), KM and KCE glazed samples, respectively.

As observed in the powders, the NIR reflectance is low in these glazed samples, with values ranging from 1.9 to 5.0% for K glazed with an electric kiln and KCE fired at 1100 °C, respectively. Likewise, their absorbance and emittance are high (Table 2), and the cut-off wavelength is not sharply defined, with a soft bandgap between 0.87 eV for K fired only with microwaves and 1.00 eV for KCE fired at 1000 °C, which represents a deficiency for its application as SSA.

### 3.3. Characterization of Glazed Samples in Double-Firing Glaze at 1050 °C: Effect of Addition of Colour Modifiers (Quartz and Anatase)

Figure 6 and Table 2 show the results obtained for the KCE sample fired in electric kiln at 1000 °C for 3 h, with 5 wt.% of the pigment incorporated into the glazes described in Section 3.1. As previously discussed, glazing the KCE in the double-firing frit at 1000 °C results in an intense black colour that reflects 98.6% of visible light. However, in the 5 wt.% glazed in double-firing glaze at 1050 °C the colour presents a blueish hue (L*a*b* = 44.2/−4.8/−8.9), suggesting a partial exsolution of copper from the pigment. It is well-established in the literature that Cu^2+^ solved in glazes can produce either a green colour, associated with octahedrally coordinated Cu^2+^ in the glassy matrix, or a blue colour, linked to square planar coordinated Cu^2+^ ions surrounded by oxygens of the glass [29]. Therefore, in this case, the Cu^2+^ exsolved in the glaze predominantly exhibit square planar coordination.

**Figure 8:**
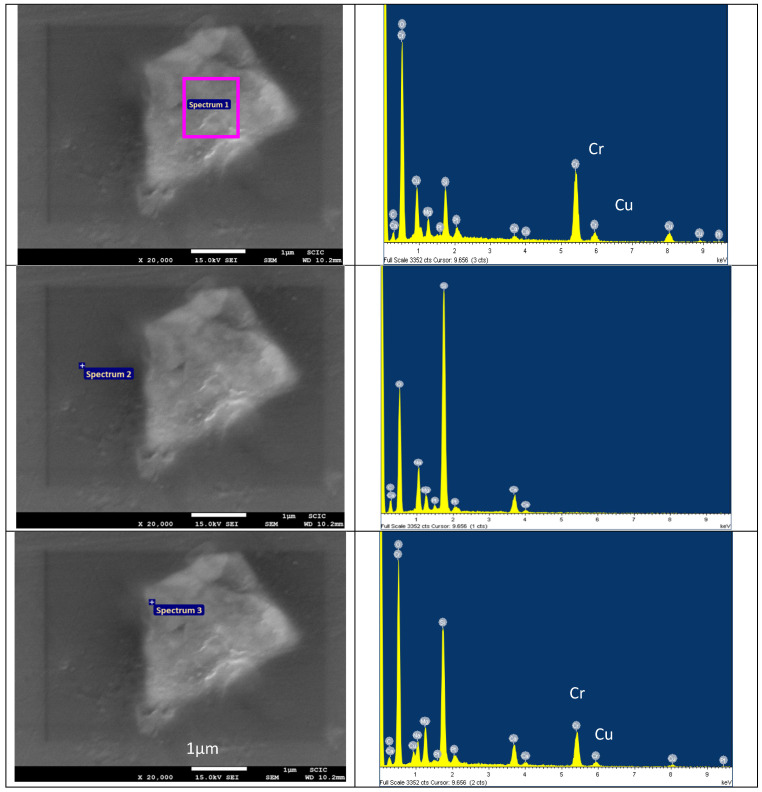
SEM image of one particle of the KCE powder calcined at 1000 °C for 3 h which was 5 wt.% glazed in the glaze at 1000 °C and the corresponding EDS analysis.

Indeed, ceramic pigments undergo an interaction with molten glass in ceramic glazes, leading to the diffusion of some ions from the ceramic particles into the glaze. The extent of this diffusion depends on the stability of the pigment and the aggressiveness of the glaze. Figure 8 presents an SEM image of a KCE powder particle calcined at 1000 °C for 3 h and incorporated at 5 wt.% into the glaze, which was fired at 1000 °C. The EDS analysis of the particle’s bulk (EDS spectrum 1), immersed in the glaze, primarily detects Cu and Cr from the pigment, along with some Ca and Na from the glaze. In the bulk glaze (EDS spectrum 2), the Cu and Cr signals disappear. Finally, at the interface (EDS spectrum 3), the intensity of the Cu and Cr signals is low, indicating slight ion diffusion into the glaze.

This instability of the pigments, which are partially dissolved by the molten glaze, is a phenomenon that is well-documented in the literature [12]. Specifically, the glaze reacts with the CuCrO_2_ pigment to form highly stable spinels, including zincochromite spinel (ZnCr_2_O_4_), which crystallizes through a reaction between the pigment and Zn^2+^ ions of the glaze:

Zn^2+^_(glaze)_ + 2CuCrO_2_ → ZnCr_2_O_4_ + 2Cu^2+^_(glaze, blue/green shade)_ (7)

The absence of Al^3+^ and Zn^2+^ in the double-firing glaze at 1000 °C inhibits the production of spinel, ensuring that the pigment remains stable in this glaze. Similarly, the low amount of Zn in porcelain glaze at 1190 °C prevents the perception of blue, even though the green parameter (b* = −2.5) is relatively high. Additionally, some pinhole defects are observed, which are likely associated with the partial reduction in exsolved Cu^2+^ to Cu^+^ [8]. This reduction releases oxygen, causing the formation of the observed pinholes:

2CuO_(glaze/green shade)_ → Cu_2_O_(glaze, red shade)_+ ½ O_2_ (8)

**Figure 9:**
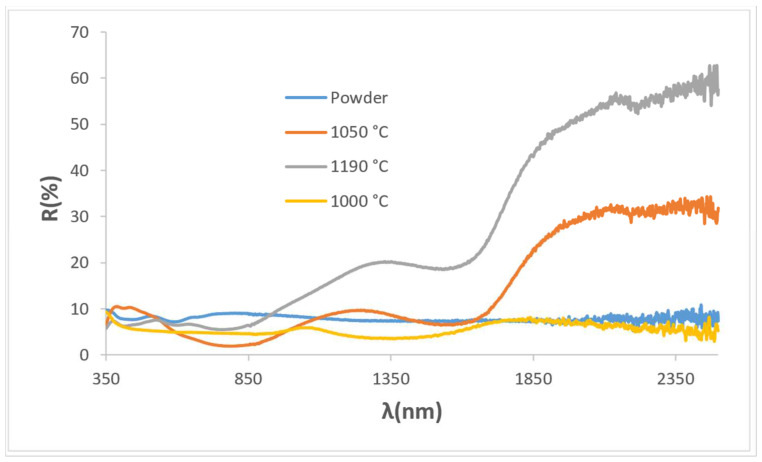
UV-Vis-NIR diffuse reflectance spectra of KC powder fired at 1100 °C and its glazed samples in 1000, 1050, and 1190 °C frits.

Figure 9 shows the UV-Vis-NIR diffuse reflectance spectra of KCE powder fired at 1100 °C and the glazed samples in frits at 1000, 1050, and 1190 °C. A sharp contrast can be observed between (1) the powder and the sample glazed with double-fired frit at 1000 °C, both of which exhibit high absorbance across the entire wavelength range, and (2) the samples glazed with frit fired at 1050 °C and porcelain frit at 1190 °C, both of which display a sharp reflectance cut-off wavelength. This behaviour, observed in cuprates [12], can be associated with the particular scattering of light by the pigment–glaze composite, which shows an inflexion point at 1725 nm. This inflexion point is related to a change in the refractive index of these glazed samples as will be discussed later. This inflexion point suggests a low bandgap of 0.59 and 0.63 eV (as well as a low emittance of 0.69 and 0.49) for the samples glazed with frit fired at 1050 °C and porcelain frit at 1190 °C, respectively. This finding indicates a promising and excellent potential for application as SSA [12].

To address pigment instability in ZnO-containing glazes, a composite effect based on dry powder coating (DPC) was considered. This method uses reflective particles (1–500 µm) as ‘hosts’, which are mechanically coated with chromophore-bearing particles (0.1–50 µm) as ‘guests’ to enhance properties such as wettability, solubility, and reactivity of the guest particles [30,31].

**Figure 10:**
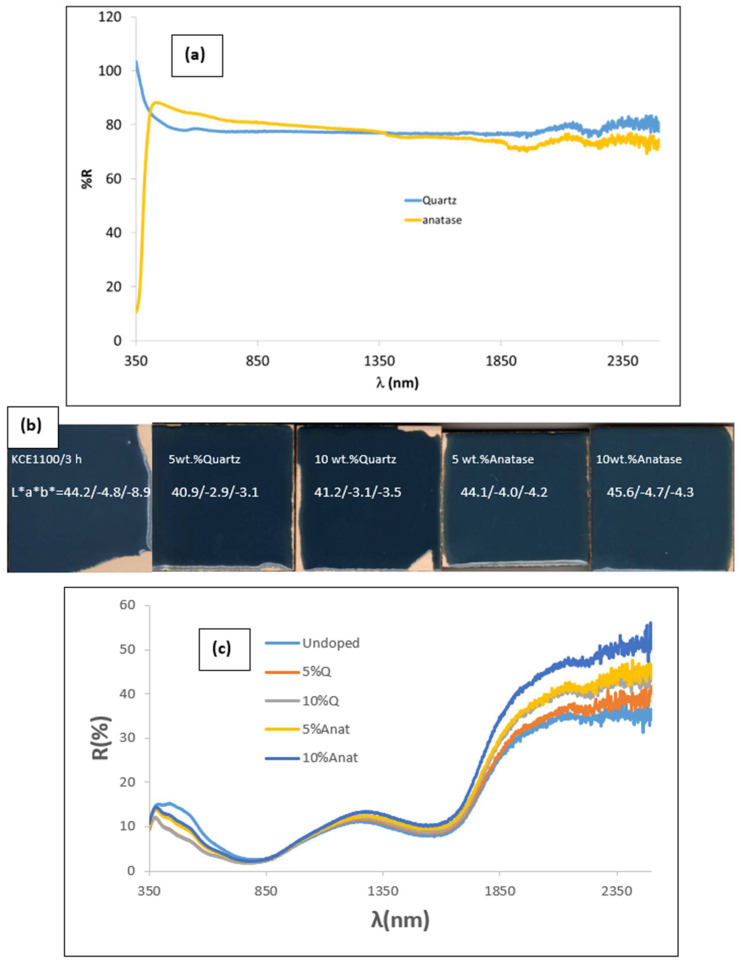
Effect of composite of quartz (Q) and anatase (Anat) with CuCrO_2_ at 1100 °C/3 h: (**a**) UV-Vis-NIR reflectance of quartz and anatase employed (supplied by QUIMIALMEL SL), (**b**) CIEL*a*b* and reflectance values of 5 wt.% of mixture glazed in the double-firing glaze at 1050 °C compared with undoped pigment, (**c**) UV-Vis-NIR diffuse reflectance spectra of glazed samples.

Figure 10 shows the results of the characterization of the composite of mcconnellite as the pigment guest (KCE fired at 1100 °C for 3 h) and quartz (Q) or anatase (Anat) as the host’s supporting particles. For this process, 10 g of mcconnellite was mechanically mixed with the corresponding amounts of quartz or anatase using an electric grinder at 20,000 rpm for 5 min. The resulting mixtures were 5 wt.% glazed in the double-firing glaze at 1050 °C.

Figure 10a shows the UV-Vis-NIR reflectance of quartz and anatase (supplied by QUIMIALMEL SL, Castelló de la Plana, Spain), both exhibiting high reflectance in the Vis and NIR ranges, around 80% in both cases. Figure 10b shows the CIEL*a*b* and reflectance values of 5 wt.% composites glazed in the double-firing glaze at 1050 °C compared with undoped pigment. Table 3 summarizes the colour and reflectance characteristics of composites.

The addition of quartz inhibits the blueish shade of the glaze, resulting in lower L*, lower C, and lower R_Vis_ compared to the unmodified sample (mcconnellite KCE fired at 1100 °C), thereby producing a better black colour. In contrast, the addition of anatase leads to higher lightness (L*) and higher R_Vis_. Although C is lower than the unmodified sample, the shade of the glaze remains similar to that of the unmodified sample. In effect, the deviation of colour of samples with quartz addition from the carbon black powder shade is smaller than of the unmodified sample (ΔE = 21.2 and 21.6 for 5%Q and 10%Q, respectively, compared to 26 for the unmodified sample). Conversely, the colour deviation of samples with anatase addition is equal to or greater than that of the unmodified sample (ΔE = 24.5 and 26.2 for 5%Anat and 10%Anat, respectively) (Table 3).

**Figure 11:**
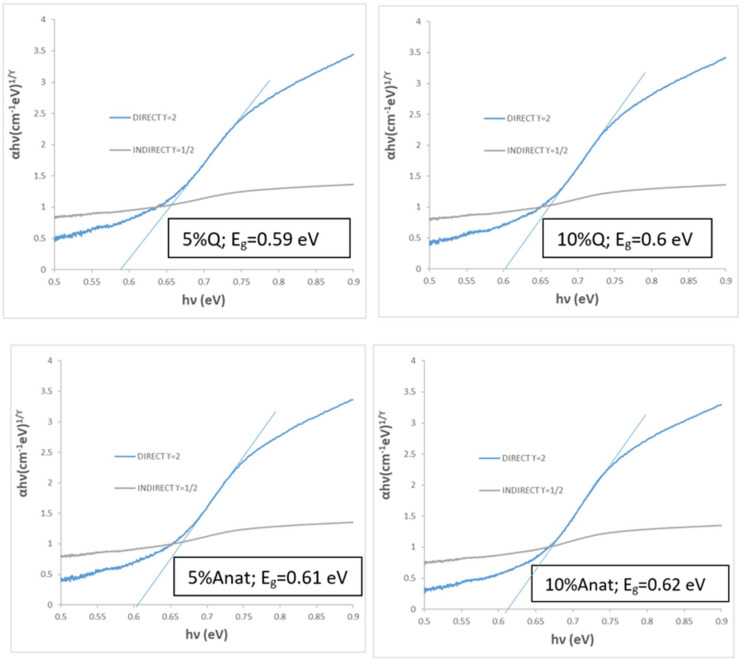
Bandgap evaluation by Tauc method of composites with quartz (Q) and anatase (Anat) with CuCrO_2_ 1100 °C for 3 h.

Figure 10c shows UV-Vis-NIR diffuse reflectance spectra of glazed samples. In accordance with colour analysis, all modified samples exhibit lower reflectance in the visible range (indicating better black colour) and higher reflectance in the NIR range, as indicated by the R_NIR_ values in Table 3. As with the unmodified parent sample, all glazed samples display a sharp cut-off wavelength between high absorptance and high reflectance (and relatively low emittance), with an inflexion point around 1725 nm. The bandgap for these samples ranges between 0.59 and 0.62 eV (Figure 11), associated with the cut-off wavelength at ~1725 nm (Figure 10c), highlighting their potential for excellent performance as selective solar absorbers (SSA).

### 3.4. Doping Effect with Lanthanide Oxides (CuCr_0.9_Ln_0.1_O_2_, Ln = La, Ce, Pr)

To address the instability of the pigment associated with a partial reduction in exsolved Cu^2+^ to Cu^+^, which releases oxygen and results in the formation of pinholes in the porcelain glaze at 1190 °C, the doping effect with lanthanide cations substituting chromium in the mcconnellite structure has been studied [32,33,34,35]. The required amount of each lanthanide oxide was used to prepare 10 g of CuCr_0.9_Ln_0.1_O_2_ (Ln = La, Ce, Pr) compositions, using copper oxide, eskolaite, and the corresponding lanthanide oxides, as described in the experimental section. The precursor mixture was mechanically homogenized in an electric grinder at 20,000 rpm for 5 min. The resulting mixtures were fired in an electric kiln at 1100 °C for 3 h. The resulting powders were 5 wt.% glazed in the porcelain single-firing glaze at 1190 °C.

**Figure 12:**
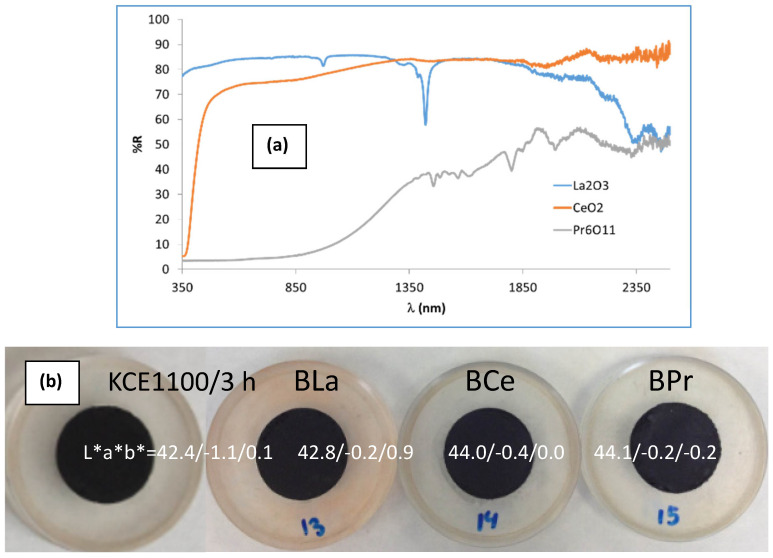

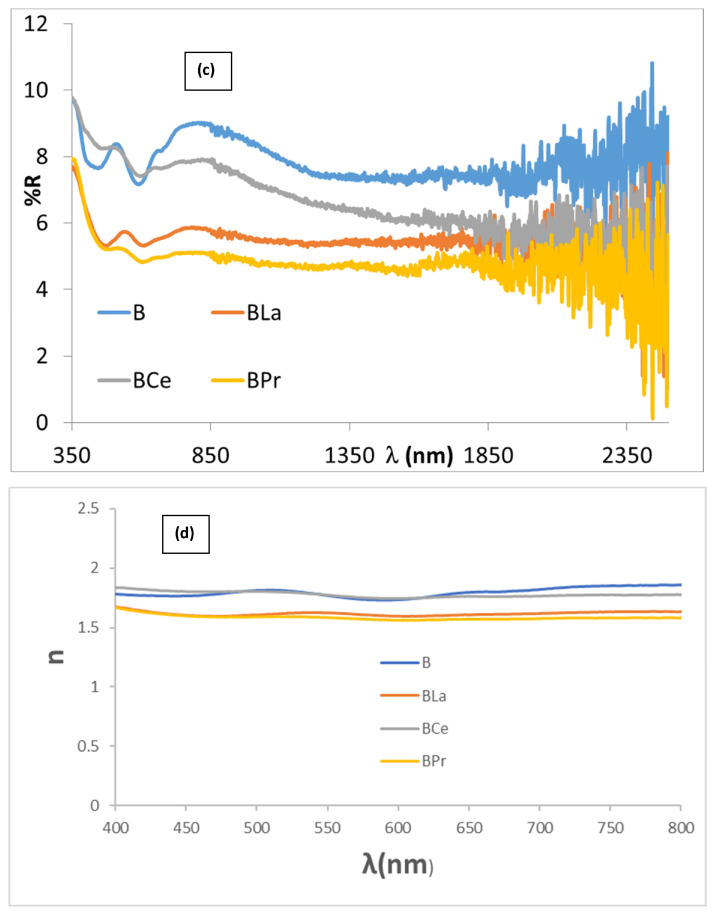

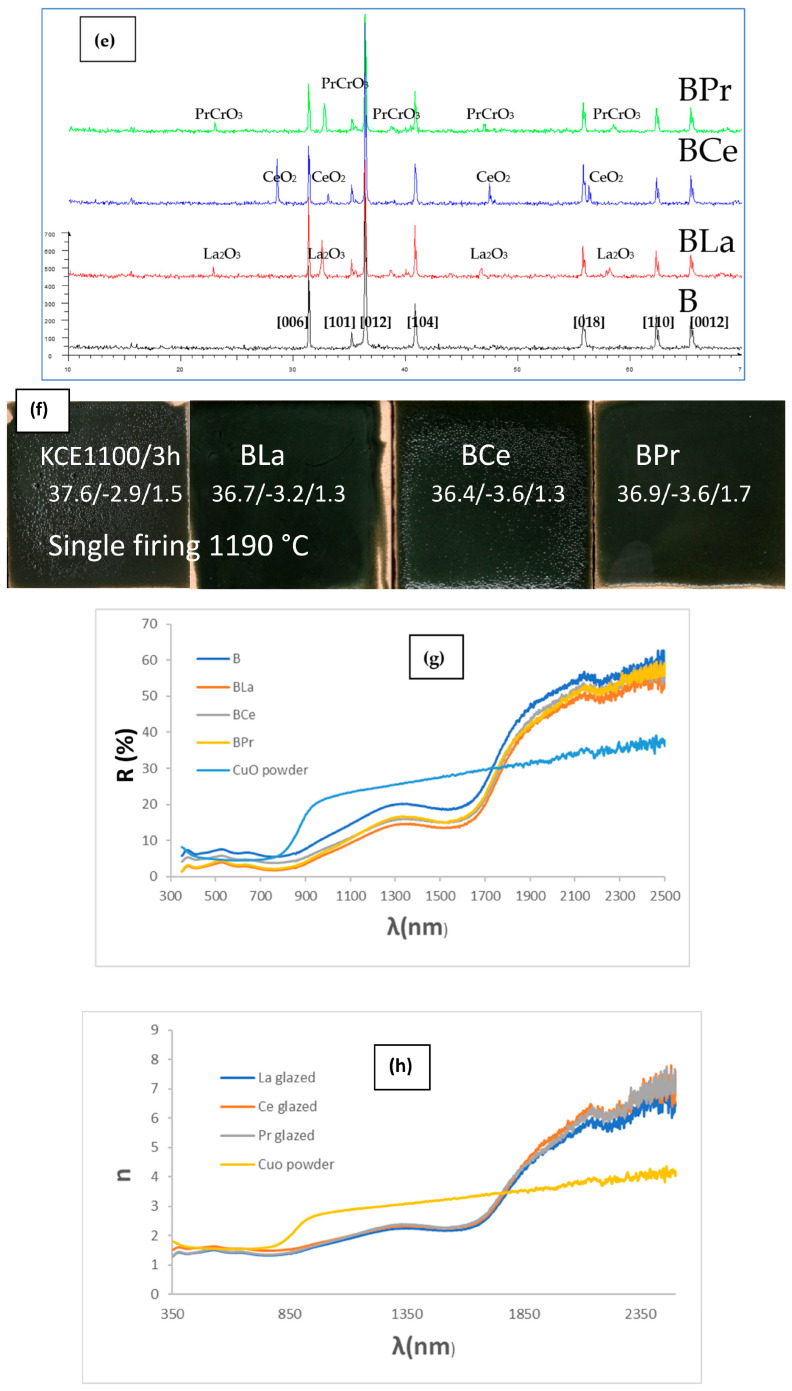
Doping effect with CuCr_0.9_Ln_0.1_O_2_ lanthanides, at 1100 °C for 3 h: (**a**) UV-Vis-NIR reflectance spectra of rare earth oxides used (supplied by ALDRICH SA), (**b**) image and CIEL*a*b* of CuCr_0.9_Ln_0.1_O_2_-fired powders, (**c**) UV-Vis-NIR reflectance spectra of CuCr_0.9_Ln_0.1_O_2_-fired powders, (**d**) refraction index of the corresponding powders in (**c**), (**d**) refraction index of samples c, (**e**) XRD diffractograms of doped samples compared with the undoped sample (B) (with hkl indexes of JCPDS 39-0247) and of CuCr_0.9_Ln_0.1_O_2_ fired powders, (**f**) CIEL*a*b* and reflectance values of 5 wt.% glazed samples in the double-firing glaze at 1190 °C compared with CuO powder (Table 2), (**g**) UV-Vis-NIR diffuse reflectance spectra of glazed samples compared with CuO powder (Table 2), (**h**) refraction index of glazed samples g compared with CuO powder (Table 2).

Figure 12a shows the UV-Vis-NIR reflectance of the rare earth oxides used (supplied by ALDRICH SA, (Darmstad, Hesse, Germany)). White oxides, such as La_2_O_3_ and CeO_2_, exhibit high reflectance across the entire light spectrum, with a total reflectance above 70%. In contrast, black Pr_6_O_11_ demonstrates low reflectance in the visible range (R_Vis_ ≈ 5%) and around 30% reflectance in the NIR region (R_NIR_), highlighting its solar selective absorber (SSA) properties.

**Figure 13:**
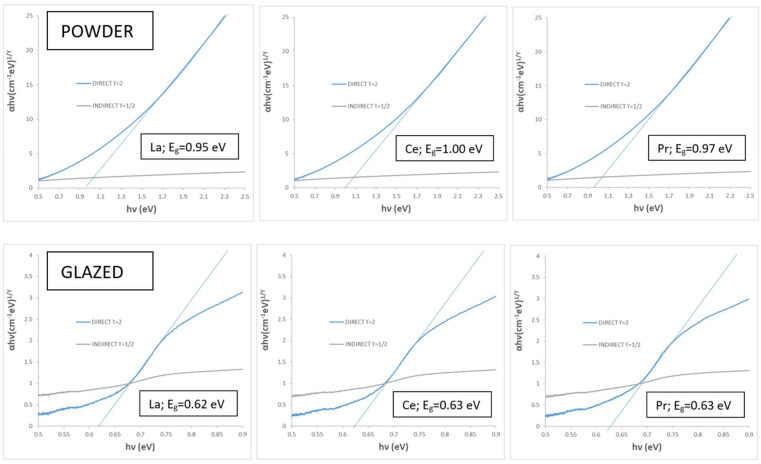
Tauc plots for doped lanthanides samples CuCr_0.9_Ln_0.1_O_2_, 1100 °C for 3 h.

Figure 12b and Table 4 present the CIEL*a*b* and reflectance data for CuCr_0.9_Ln_0.1_O_2_ fired powders, compared with the undoped pigment. The results show that the doped samples exhibit lower (C) and reduced visible reflectance (R_Vis_) compared to the unmodified sample (mcconnellite KCE fired at 1100 °C). However, the lightness (L*) and the deviation (ΔE) from carbon black values increase, indicating that the doped pigments produce a similar (in the case of Ce) or slightly improved black coloration (in the case of La and Pr) compared to the undoped black powder (Table 4). In effect, the UV-Vis-NIR reflectance spectra of CuCr_0.9_Ln_0.1_O_2_ fired powders (Figure 12c) confirm these findings. The Ce-doped sample exhibits a visible reflectance (R_Vis_), similar to the undoped sample (8.0% and 8.1%, respectively, as shown in Table 4). In contrast, the La- and Pr-doped samples display lower visible reflectance values (R_Vis_ = 5.6% and 5.2%, respectively, as detailed in Table 4). The band gap of the doped samples ranges between 0.95 and 1.0 eV (Figure 13), lower than the corresponding undoped samples (e.g., 1.31 eV, KCE sample fired at 1100 °C for 3 h, Table 2), indicating the effect of lanthanide addition.

Figure 12d. presents the estimated refractive index (*n*) of the samples in the visible range, derived from the corresponding reflectance spectra in Figure 12c using the previously discussed Fresnel equation. The refractive index of the undoped and Ce-modified samples is slightly higher than that of the La- and Pr-modified powders, indicating that they act as slightly better opacifiers in glazes.

Figure 12e shows the XRD diffractograms of doped samples compared to the undoped sample. In all cases, diffraction peaks corresponding to non-reacted lanthanide phases can be detected (La_2_O_3_ and CeO_2_ for La- and Ce-doped samples and PrCrO_3_ for the Pr-doped sample). These phases act as composite modifiers.

The synthesized mcconnellite has been indexed as the rhombohedral delafossite phase. The cell parameters and the volume (V) of the unit cell were calculated using the 2θ positions of the diffraction peaks, applying Equations (9) and (10) [36,37]:

(9) $$\frac{1}{d^{2}}=\frac{1}{a^{2}}(\frac{{(h}^{2}+hk+k^{2})\left(1+{cos}^{2}\alpha \right)-(hk+kl+lh)(1-{tan}^{2}\frac{\alpha }{2})}{1+cos\alpha {-2cos}^{2}\alpha })$$

(10) $$V=a^{3}{\left(1-3cos\alpha {+2cos}^{2}\alpha \right)}^{1/2}$$

where *d* is the interplanar distance of the respective (hkl) lattice plane, obtained from °2θ using Bragg’s law, and a = b = c represents the unit cell edges and α the rhombohedral angle. The results are summarized in Table 5, showing a slight increase in cell dimensions, likely due to the incorporation as solid solution of the lanthanide ions with higher ion size (crystal radius of Shannon: 1.172, 1.150 and 1.130 Å for La^3+^, Ce^3+^ and Pr^3+^ ions, respectively, compared to 0.755 Å for Cr^3+^ in octahedral coordination) [38]. Both the solid solution and the composite effect may contribute to the enhanced visible light absorbance observed in the lanthanide-modified samples compared to the undoped pigment.

Figure 12f and Table 4 show the CIEL*a*b* values and reflectance data of 5 wt.% glazed CuCr_0.9_Ln_0.1_O_2_ powders in the single-firing porcelain glaze at 1190 °C, compared with the undoped pigment. The results indicate that doped samples exhibit lower lightness (L*), lower chroma (C), and reduced visible reflectance (R_Vis_) compared to the unmodified sample (mcconnellite KCE fired at 1100 °C). Additionally, the deviation from carbon black (ΔE) decreases, resulting in a better black coloration than the undoped black powder (Table 4).

Furthermore, the UV-Vis-NIR reflectance spectra of CuCr_0.9_Ln_0.1_O_2_-fired powders, compared with the CuO powder from Table 2 (Figure 12g), reveal that the reflectance across the entire light range is lower for the modified samples than for the undoped pigment. Specifically, the RVis values are 2.9%, 4.9%, and 3.3% for the La-, Ce-, and Pr-doped samples, respectively, compared to 6.3% for the glazed undoped pigment (Table 4).

The band gap of the glazed doped samples in the frit fired at 1190 °C ranges between 0.62 and 0.63 eV (Figure 13), similar to that of the corresponding undoped samples (e.g., 1.63 eV for the KCE sample in the frit fired at 1190 °C, Table 2). The cut-off wavelength intensity is higher and occurs at a longer wavelength than that of the CuO powder used as an SSA reference. Similarly, the refractive index of the samples (Figure 12h) shows the cut-off wavelength with higher intensity and at a longer wavelength.

On the other hand, La and Pr effectively inhibit the pin-hole defects observed in the undoped pigment. However, the Ce-modified sample still shows the presence of pinhole defects. Therefore, some stabilization of copper can be detected on La- and Pr-modified samples. This suggests some stabilization of copper in the La- and Pr-modified samples.

## 4. Conclusions

Mcconnellite CuCrO_2_ was synthetized using both the solid-state and dielectric calcination using microwaves and has been characterized as a novel black ceramic pigment in different industrial glazes. The pigment prepared with microwave assistance (60 min at 800 W) exhibits a deeper black colour compared to the pigment obtained through electric firing at 1000 °C for 3 h. This is demonstrated by its lower deviation (ΔE*) from carbon black, which was used as a reference, and its lower visible reflectance (R_Vis_: 15.7% and 9.7% versus 23.1% and 13.8%, respectively), correlating with its smaller particle size and optimized microstructure. When microwave-assisted pigment is glazed in a double-firing glaze at 1000 °C using an electric kiln, it produces a black shade closer to carbon black (ΔE* = 3.7) compared to conventional glazing with an electric kiln (ΔE* = 9.5). This result surpasses the performance of the reference carbon black powder, absorbing 98.6% of visible light.

Both powders and glazed samples in the double-firing glaze at 1000 °C show low NIR reflectance values ranging from 1.9 (for K glazed with an electric kiln) to 5.0 (for KCE fired with electric kiln at 1100 °C). Their absorbance and emittance are high, with an unsharpened cut-off wavelength, which presents a limitation for their application as solar selective absorbers (SSA).

Using higher maturation glazes, the newly developed mcconnellite pigments exhibit a bluish hue in double firing at 1050 °C, which is associated with the presence of zinc. Furthermore, pinhole defects are observed in the porcelain single-firing glaze at 1190 °C, associated with the destabilization of copper (I). For glazed samples with a double-firing glaze at 1050 °C and a porcelain glaze at 1190 °C, a sharp cut-off wavelength is observed between high absorptance and high reflectance, along with low emittance. This cut-off, with an inflexion point at 1725 nm, highlights their excellent potential for application as SSAs.

Composites of the fired pigment with quartz suppress the blueish shade, producing a better black colour when glazed in the double-firing glaze at 1050 °C. In contrast, composites with anatase exhibit a glazing behaviour, similar to the unmodified sample.

Doped samples with formal stoichiometry CuCr_0.9_Ln_0.1_O_2_ (Ln = La, Ce or Pr) show improved black colour compared to the undoped black powder (R_Vis_ = 2.9, 4.9 and 3.3%, respectively, versus 6.3% for unmodified pigment). Furthermore, La and Pr inhibit the pin-hole defects observed in the undoped pigment. However, pinhole defects persist in the Ce-modified sample. This suggests that La and Pr modifications contribute to the stabilization of copper in the samples.

## Figures and Tables

**Table 1:** Compositions by EDS estimation of used frits (supplied by Torrecid SA, l’Alcora, Spain).

	Double Firing 1000 °C	Double Firing 1050 °C	Single Firing 1190 °C
**Oxide**			
SiO_2_	72	59	67
Na_2_O	14	4	-
K_2_O	1.5	5	3
CaO	9	15	12.5
MgO	-	-	1.5
ZnO	-	9	6
Al_2_O_3_	2.3	8	10
PbO	-	-	-

**Table 2:** Colour and reflectance characteristics of samples (values are displayed with the last safe significant digit, e.g., 2.8 is 2.8 ± 0.1 or 99 is 99 ± 1).

Sample	L*a*b*	R_Vis_/R_NIR_/R (%)	ΔE*	C*	h	α	ε_100_	E_g_ (eV)
***Powders***								
**K** microwaves firing	36.5/−4.8/−0.8	9.9/11.2/10.5	17.0	4.9	189.5	0.90	0.88	1.30
**KM** microwaves firing plus 1000 °C/3 h	35.1/−4.8/0.1	9.7/11.8/10.6	15.7	4.8	178.8	0.89	0.87	1.28
**KCE** electric kiln **T(°C)/3 h**								
1000/3 h	42.6/−5.4/0.1	13.8/14.1/13.9	23.1	5.4	178.9	0.86	0.85	1.26
1100/3 h	42.4/−1.1/0.1	8.1/8.2/8.0	22.2	1.1	175.0	0.93	0.91	1.31
1200/3 h	44.5/−1.2/1.7	8.9/14.6/11.7	24.4	2.1	125.2	0.88	0.82	1.30
***Glazed***								
**K** glazed with microwaves (Glaze 1000)	23.5/−1.6/−0.4	3.9/6.2/4.9	3.7	1.7	166.0	0.95	0.92	0.87
**K** glazed with electric kiln (Glaze 1000)	11.3/−0.6/−3.0	1.4/1.9/1.6	9.5	3.1	78.7	0.98	0.96	0.93
**KM** (Glaze 1000)	11.8/−0.6/−2.5	1.5/2.2/1.8	8.8	2.6	76.5	0.98	0.96	0.95
**KCE**								
Glaze 1000	26.9/−0.1/−2.3	5.3/5.0/5.2	7.2	2.3	87.5	0.95	0.94	1.00
Glaze 1050	44.2/−4.8/−8.91	6.3/7.5/6.9	26.0	10.1	118	0.91	0.69	0.59
Glaze 1190	37.6/−2.9/1.5	6.7/15.9/11.2	17.7	3.3	27.4	0.89	0.49	0.63
**CuO**	25.8/0.3/−2.4	5.1/21.2/12.2	6.3	2.41	83.1	0.89	0.69	1.40
**Carbon Black** (powder)	20.2/0.1/0.1	3/3/3		0.14	45	0.99	0.96	1.40

**Table 3:** Colour and reflectance characteristics of composites of KCE 1100 °C for 3 h with quartz (Q) or anatase (Anat) glazed in double-firing glaze at 1050 °C (values are displayed with the last safe significant digit, for example, 2.8 is 2.8 ± 0.1 or 99 is 99 ± 1).

Sample	L*a*b*	R_Vis_/R_NIR_/R (%)	ΔE*	C*	h	α	ε_100_	E_g_ (eV)
Glazed 1050	44.2/−4.8/−8.91	6.3/7.5/6.9	26.0	10.1	118	0.91	0.69	0.59
5 wt.% Q	40.9/−2.9/−3.1	5.7/9.0/7.3	21.2	4.2	46.0	0.92	0.66	0.59
10 wt.% Q	41.2/−3.1/−3.5	5.6/9.4/7.5	21.6	4.7	48.5	0.92	0.63	0.60
5 wt.% Anat	44.1/−4.0/−4.2	7.1/9.8/8.4	24.5	5.8	46.4	0.92	0.62	0.61
10 wt.% Anat	45.6/−4.7/−4.3	7.6/10.6/9.1	26.2	6.4	42.5	0.91	0.56	0.62

**Table 4:** Colour and reflectance characteristics of composite effect of lanthanide stoichiometric addition CuCr_0.9_Ln_0.1_O_2_, 1100 °C for 3 h (values are displayed with the last safe significant digit, for example, 2.8 is 2.8 ± 0.1 or 99 is 99 ± 1).

Sample	L*a*b*	R_Vis_/R_NIR_/R (%)	ΔE*	C*	h	α	ε_100_	E_g_ (eV)
Undoped powder	42.4/−1.1/0.1	8.1/8.2/8.0	22.2	1.1	175.0	0.93	0.91	1.31
La	42.8/−0.2/0.9	5.6/5.5/5.6	22.6	0.9	102.5	0.94	0.95	0.95
Ce	44.0/−0.4/0.0	8.0/7.0/7.5	23.8	0.4	180.0	0.93	0.95	1.00
Pr	44.1/−0.2/−0.2	5.2/4.8/5.0	23.9	0.3	135.0	0.95	0.96	0.97
Glazed 1050	44.2/−4.8/−8.91	6.3/7.5/6.9	26.0	10.1	118.0	0.91	0.69	0.59
La	36.7/−3.2/1.3	2.9/11.2/7.0	13.9	3.5	158.0	0.93	0.54	0.62
Ce	36.4/−3.6/1.3	4.9/12.9/8.8	16.7	3.8	160.1	0.91	0.51	0.63
Pr	36.9/−3.6/1.7	3.3/12.4/7.7	17.2	4.0	154.7	0.92	0.52	0.63

**Table 5:** Cell parameters and the volume V of the cell of lanthanide stoichiometric addition CuCr_0.9_Ln_0.1_O_2_, 1100 °C for 3 h (values are displayed with the last safe significant digit, for example, 2.8 is 2.8 ± 0.1 or 99 is 99 ± 1).

Sample	a (Å)	α (°)	V (Å^3^)	ΔV (%)
Undoped powder	8.172	20.09	56.327	reference
La	8.281	19.81	57.032	1.2
Ce	8.207	19.97	56.432	0.2
Pr	8.195	19.99	56.271	−0.1

## Data Availability

The original contributions presented in this study are included in the article. Further inquiries can be directed to the corresponding author.

## Abbreviations

The following abbreviations are used in this manuscript:

SSA	Selective solar absorber
FPSC	Flat-plate solar collector
IR	Infrared
XRD	X-ray diffraction
SEM–EDS	Scanning electron microscopy with energy-dispersive spectroscopy
JCPDS	Joint Committee on Powder Diffraction Standards
UV-Vis-NIR	Ultraviolet–visible–near-infrared
